# Shared Genetic Background between Parkinson’s Disease and Schizophrenia: A Two-Sample Mendelian Randomization Study

**DOI:** 10.3390/brainsci11081042

**Published:** 2021-08-06

**Authors:** Kiwon Kim, Soyeon Kim, Woojae Myung, Injeong Shim, Hyewon Lee, Beomsu Kim, Sung Kweon Cho, Joohyun Yoon, Doh Kwan Kim, Hong-Hee Won

**Affiliations:** 1 Department of Psychiatry, Kangdong Sacred Heart Hospital, College of Medicine, Hallym University, Sungan-ro, Kangdong-gu, Seoul 05355, Korea; kkewni@gmail.com; 2 Samsung Medical Center, Department of Digital Health, Samsung Advanced Institute for Health Sciences and Technology (SAIHST), Sungkyunkwan University, Seoul 06351, Korea; soyeon2019@skku.edu (S.K.); injeong.shim@gmail.com (I.S.); kyce@hanmail.net (B.K.); 3 Department of Neuropsychiatry, Seoul National University Bundang Hospital, Seongnam 13620, Korea; jy3020@tc.columbia.edu; 4 Department of Health Administration and Management, College of Medical Sciences, Soonchunhyang University, Asan 31538, Korea; woniggo@gmail.com; 5 Department of Software Convergence, Graduate School, Soonchunhyang University, Asan 31538, Korea; 6 Department of Pharmacology, School of Medicine, Ajou University, Worldcup-ro, Yeongtong-gu, Suwon 16499, Korea; wontan2000@gmail.com; 7 Samsung Medical Center, Department of Psychiatry, School of Medicine, Sungkyunkwan University, Seoul 06351, Korea; paulkim@skku.edu

**Keywords:** Parkinson’s disease, schizophrenia, Mendelian randomization, genetics

## Abstract

*Background and objectives*: Parkinson’s disease (PD) and schizophrenia often share symptomatology. Psychotic symptoms are prevalent in patients with PD, and similar motor symptoms with extrapyramidal signs are frequently observed in antipsychotic-naïve patients with schizophrenia as well as premorbid families. However, few studies have examined the relationship between PD and schizophrenia. We performed this study to evaluate whether genetic variants which increase PD risk influence the risk of developing schizophrenia, and vice versa. *Materials and Methods*: Two-sample Mendelian randomization (TSMR) with summary statistics from large-scale genome-wide association studies (GWAS) was applied. Summary statistics were extracted for these instruments from GWAS of PD and schizophrenia; *Results*: We found an increase in the risk of schizophrenia per one-standard deviation (SD) increase in the genetically-predicted PD risk (inverse-variance weighted method, odds ratio = 1.10; 95% confidence interval, 1.05−1.15; *p* = 3.49 × 10^−5^). The association was consistent in sensitivity analyses, including multiple TSMR methods, analysis after removing outlier variants with potential pleiotropic effects, and analysis after applying multiple GWAS subthresholds. No relationships were evident between PD and smoking or other psychiatric disorders, including attention deficit hyperactivity disorder, autism spectrum disorder, bipolar affective disorder, major depressive disorder, Alzheimer’s disease, or alcohol dependence. However, we did not find a reverse relationship; genetic variants increasing schizophrenia risk did not alter the risk of PD; *Conclusions*: Overall, our findings suggest that increased genetic risk of PD can be associated with increased risk of schizophrenia. This association supports the intrinsic nature of the psychotic symptom in PD rather than medication or environmental effects. Future studies for possible comorbidities and shared genetic structure between the two diseases are warranted.

## 1. Introduction

Parkinson’s disease (PD) is the second most common progressive neurodegenerative disease. PD is characterized by tremors, bradykinesia, rigidity, and posture instability [1]. Motor symptoms have been the primary focus in the diagnosis and the treatment target of patients with PD. However, non-motor symptoms have recently been described and include depression, anxiety, sleep problems, bowel and bladder habit changes, autonomic disturbances, and sensory complaints. Psychiatric symptoms that cause pronounced distress in patients with PD are also common and are closely related to low life satisfaction and quality of life [2]. Anxiety symptoms in PD have been reported by up to 30% of patients [3], and are accompanied by depression, somatic symptoms, and hostility-irritability. Suicide risk in PD patients is also higher than in the general population [4,5].

Psychotic symptoms are also frequently observed non-motor symptoms of PD, with a prevalence of 20% to 70%. Symptoms of the psychosis spectrum in early PD consist of minor experiences, such as passage and presence of hallucinations, illusions, and formed hallucinations, including recurring visual hallucinations with insight preserved [6]. Delusions and hallucinations can occur in the later stage of PD [7,8]. However, the biological mechanisms of psychotic symptoms in PD—whether based on PD itself, on cognitive decline, or on medication side effect—are not clearly understood. The primary treatment of choice for motor symptoms in PD is dopamine agonist. The onset of PD psychosis is suggested by the hyper-regulation of dopamine elicited by medication, which can be relieved by dose reduction. However, there has been no evidence of a direct causal relationship between pharmacological treatments or medication dose and psychotic symptoms in PD [9]. There is still a need for further investigation to clarify the causal relationship between PD psychosis and dopamine-related pathology.

In schizophrenia, although positive psychotic symptoms and negative symptoms as deficit are the usual described core features, motor symptoms have also been recently highlighted [10]. Motor symptoms, called parkinsonian symptoms, are frequently observed both in premorbid families of schizophrenia and in naïve patients for antipsychotics [11].

Despite the similarity between observed symptomatology of PD and schizophrenia, several components, including differences in the age of onset and incompatibility in dopamine hypothesis, and possible adverse effects of medications, have hampered research on an association between PD and schizophrenia. Only a few fragmented studies on the association between PD and schizophrenia have been reported [12,13].

It is difficult to establish the genetic association between PD and schizophrenia by observational epidemiological approaches. One promising approach to investigating the association is Mendelian randomization (MR) using genetic variants as the instrumental variables [14]. Association between genetic variants and disease outcome state can provide evidence with minimizing confounding factors, including age, medication, or environmental exposures.

We hypothesized that PD and schizophrenia have shared genetic background and the risk for PD has a causal effect on the risk for schizophrenia and *vice versa*. We tested the hypothesis using two-sample MR methods with summary statistics from large-scale genome-wide association studies (GWAS) of PD and schizophrenia.

## 2. Materials and Methods

### 2.1. Datasets

For two-sample MR, we obtained summary statistics from large-scale GWAS of PD, schizophrenia, and other psychiatric diseases or related traits, including bipolar disorder, major depressive disorder, Alzheimer’s disease, alcohol dependence, and smoking. The GWASs of other psychiatric disorders or traits were used to test whether the genetic variants for PD act on schizophrenia through other biological pathways (horizontal pleiotropy) other than the direct effect of PD. They are available on the LD Hub website (http://ldsc.broadinstitute.org/gwashare/; accessed on 1 May 2020), and details are listed in Supplementary Table S1. We also used the information of recently identified single nucleotide polymorphisms (SNPs) from GWAS of PD [15]. For SNP selection, we conducted the following steps. First, we obtained SNPs as instrumental variants that were significantly associated with one disease or trait (exposure) at the P-thresholds for suggestive significance or at a stricter level (*p* < 5 × 10^−8^–5 × 10^−6^). The relaxed thresholds have been considered in previous MR studies, given an exposure GWAS with a small number of genome-wide significant SNPs (*p* < 5 × 10^−8^) such as PD GWAS in this study [16,17]. Second, to ensure that the instruments for exposure were independent of each other, we performed linkage disequilibrium (LD) clumping with a 10 Mb window size and LD value (*r*^2^ < 0.001) using data from European individuals from the 1000 Genomes Project Phase 3. Third, we extracted the same SNPs from GWAS of other diseases or traits (outcome), and by comparing the frequencies and effect sizes of the same SNPs on the exposure with those on the outcome, we removed SNPs with ambiguous alleles from the set of instruments.

### 2.2. Statistical Analyses

One of the underlying assumptions of the examination of the relationship using MR is that the instrumental variants have no pleiotropic effects. The effect estimate can be severely biased if the genetic variants extracted as instrumental variables violate this assumption. Therefore, we applied the MR-PRESSO method to detect and remove instruments with potential pleiotropic effects to eliminate the bias [18]. The MR-PRESSO consists of a global test, outlier test, and distortion test. Through a three-step procedure, we detected outlier variants having pleiotropic effects and excluded these in the subsequent analysis. We performed two-sample Mendelian randomization (TSMR) to infer the association of an exposure on an outcome using summary statistics from GWAS [19]. We performed TSMR analyses using the MR-Base software (http://www.mrbase.org/; accessed on 1 May 2020) that provides various functions for combining, harmonizing, and utilizing GWAS summary statistics. Multiple methods for TSMR have been developed and are different from each other in terms of sensitivity to heterogeneity, bias, and power. We selected the inverse-variance weighted (IVW) method as our main TSMR method because it provides reliable results in the presence of heterogeneity in an MR analysis and is appropriate when using a large number of SNPs [20,21,22]. The standard error of the IVW effect was estimated using a multiplicative random effects model. We performed leave-one-out analysis to test if the results were derived from any particular SNP. A forest plot was used to visualize heterogeneity between instruments due to horizontal pleiotropy and the contribution of each instrument to the overall estimate [19]. We also used the MR Egger regression (MR-Egger) and the weighted median (WM) for sensitivity analyses [23,24]. Since these two methods provide reliable causal estimates in the presence of a violation of MR assumptions, WM and MR-Egger have been used as sensitivity analyses in MR studies [23,25]. Since horizontal pleiotropy can be a confounding factor in MR, we tested pleiotropy by performing MR-Egger with the intercept unconstrained. The intercept of the MR-Egger shows a statistical estimate of the presence of directional pleiotropy.

## 3. Results

### 3.1. Causality between PD Risk and Schizophrenia Risk

To investigate the shared genetic background between PD risk and schizophrenia risk, we selected four genetic variants that were significantly associated with risk of PD from GWAS with 1713 cases with PD and 3978 controls as instruments (Table 1) [26]. We extracted summary statistics for these instruments from GWAS of schizophrenia in the Psychiatric Genomics Consortium (PGC) with 35,476 cases and 46,839 controls [27]. The same alleles of the four SNPs increased both the risk of PD and the risk of schizophrenia. We annotated the four instrumental variants using Variant Effect Predictor (VEP) [28]. Three of these SNPs were located at the *CDH8*, *SNCA,* and *WNT3* genes, with no known gene near the other SNP.

**Figure 1:**
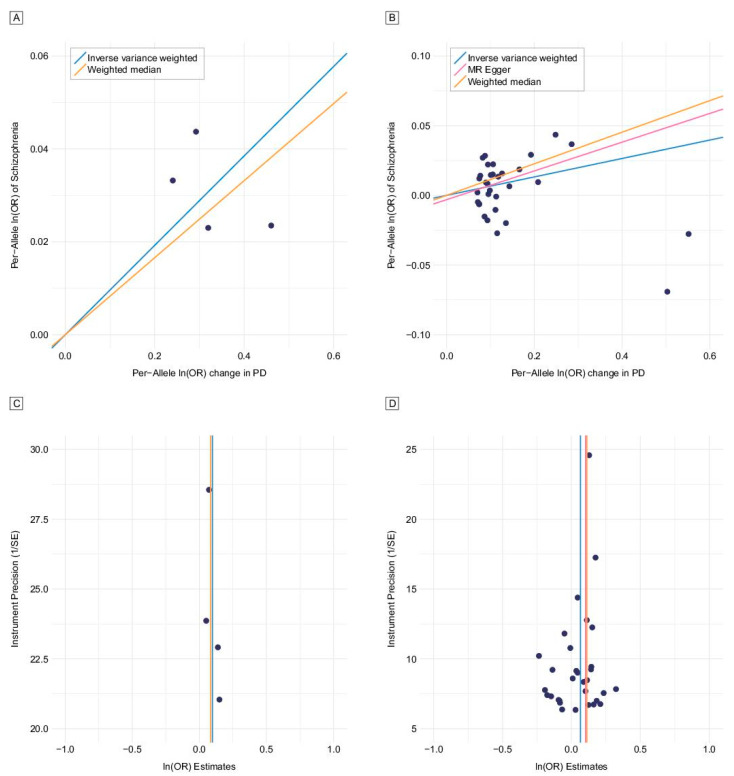
Mendelian randomization analysis for risk of Parkinson’s disease (PD) on schizophrenia using genetic instruments. (**A**) Schizophrenia associations (scatter plot) using four genetic instruments (PD) [24]. (**B**) Schizophrenia associations (scatter plot) using 32 genetic instruments (PD) [15]. (**C**) Precision (funnel plot) using four genetic instruments (PD) [24]. (**D**) Precision (funnel plot) using 32 genetic instruments (PD) [15].

**Figure 2:**
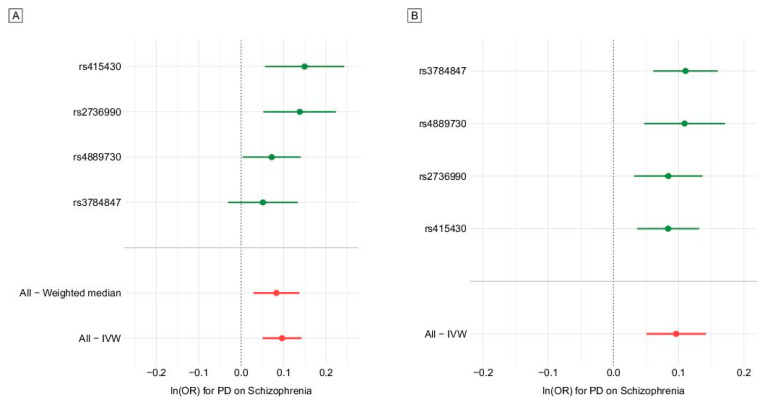
Forest and leave-one-out plots showing association with schizophrenia for each genetic instrument of Parkinson’s disease (PD). (**A**) Forest plot using four genetic instruments (PD) [24]. (**B**) Leave-one-out plot using four genetic instruments (PD) [24].

Two-sample MR using IVW revealed a causality of PD risk on schizophrenia risk (odds ratio [OR] per log odds increase in PD risk 1.10, 95% confidence interval [CI] 1.05–1.15, *p* = 3.49 × 10^−5^) (Figure 1A and Table 2). A funnel plot was constructed. In a funnel plot, each dot shows the proportion of the precision (1/standard error) to Wald ratios per SNP, and the vertical line indicates the MR estimates jointed by the four instruments (Figure 1C). We observed overall symmetry in the funnel plot (asymmetry represents heterogeneity driven by directional horizontal pleiotropy that violates MR assumptions) [19]. Even after relaxing the *p*-value threshold for defining the instruments for exposure and including more SNPs with less significant association with PD risk, a causal effect of PD on schizophrenia risk remained significant (Table 2). OR, 95% CIs, and *p*-values of IVW method are shown in Supplementary Table S2. The Cochrane *Q* statistics, *Q* value, and heterogeneity (*I*^2^[%]) were 2.54, 0.47, and 0, respectively, which indicated little heterogeneity between instrumental variants in the MR analysis for the risk of PD (Figure 2A). Leave-one-out analysis showed that all the SNPs contributed to the association of PD with schizophrenia (Figure 2B). In contrast, TSMR analysis with schizophrenia risk as exposure and PD risk as outcome showed no evidence for the causal effect of schizophrenia risk on PD risk (Supplementary Tables S3 and S4).

In addition, we performed sensitivity analysis using WM and tested horizontal pleiotropy using MR-Egger (Supplementary Tables S2 and S5). The effect of PD on the risk of schizophrenia was significant in the WM analysis (OR 1.09, 95% CI 1.02–1.15, *p* = 3.77 × 10^−3^). MR-Egger showed no evidence of horizontal pleiotropy (intercept OR 1.05, 95% CI 0.99–1.11, *p* = 0.25).

We also tested newly discovered SNPs in a recent GWAS study with 6476 cases with PD and 302,042 controls [15]. The data included 34 SNPs associated with PD at the genome-wide significance level. Since two of the 34 variants showed pleiotropy when applying the outlier test of MR-PRESSO, we excluded them in the MR analysis. The IVW method using the 32 SNPs showed a positive causal effect (OR 1.07, 95% CI 1.02–1.12, *p* = 1.81 × 10^−3^) on schizophrenia risk per log odds increase in PD risk (Figure 1B,D). The effect of PD risk on the risk of developing schizophrenia was significant in IVW (*p* = 1.81 × 10^−3^) and WM (*p* = 2.84 × 10^−5^), but was marginally significant in MR-Egger (*p* = 0.05). However, both IVW and MR-Egger results were significant for SNPs passing GWAS sub-thresholds of *p* < 5 × 10^−7^ and *p* < 5 × 10^−6^ (Supplementary Tables S6 and S7). There was moderate heterogeneity between instrumental variants in the MR analysis for the risk of PD; the Cochran *Q* statistics, *Q* value, and heterogeneity (*I*^2^[%]) were 45.43, 0.05, 32, respectively (Supplementary Figure S1A). However, the result of leave-one-out analysis for the risk of PD showed that single SNPs were not exclusively responsible for the associations of the risk of PD (Supplementary Figure S1B). MR-Egger suggested no evidence of pleiotropy (intercept OR 1.00, 95% CI 0.98–1.01, *p* = 0.49).

### 3.2. Shared Genetic Background between PD and Other Psychiatric Disorders or Related Traits

We performed TSMR for other psychiatric disorders to investigate if PD also has a shared genetic background with any other psychiatric disorders or related traits than schizophrenia. The other eight psychiatric disorders or related traits were not related to PD in the IVW MR results (Table 2). This implies that the SNPs selected as instruments showed no pleiotropy within the psychiatric traits. Even after mitigating the GWAS *p*-value threshold to define additional instrumental variants for PD, the association of PD risk on the seven psychiatric traits was not significant (Table 2). Our analysis showed no evidence for shared genetic background between PD and other psychiatric disorders.

## 4. Discussion

We used TSMR to evaluate the association between PD and schizophrenia. Our findings support a possible shared genetic background between PD and schizophrenia. The genetic variants increasing the risk of PD were likely to influence the increased risk of schizophrenia. However, we did not observe a reverse directional relationship; genetic variants increasing schizophrenia risk did not increase the risk of PD.

The relationship between PD and schizophrenia was significant in the IVW and WM analyses, but not in the MR-Egger regression approach. Of note, the causal inference by MR can be severely affected if a fundamental assumption of “no pleiotropy” is not satisfied. MR-Egger was developed to detect violations of this assumption and to provide a robust effect estimate when genetic instrumental variables showed pleiotropy. Our MR-Egger analyses suggested that there was no clear evidence for an influence of biological pleiotropy on the findings [23]. Because the power of MR-Egger was previously shown to be lower than other conventional methods, we used the IVW as the main MR method and the WM for sensitivity analysis. In addition, we also used the latest large-scale PD GWAS in 2017 [15] including more individuals than PD GWAS in 2009. By including more genetic variants and removing outlier variants with potential pleiotropic effects using MR-PRESSO, the effect of genetic risk of PD on the risk for schizophrenia appeared to be significant in the MR-Egger analysis. Moreover, our analysis using multiple GWAS subthresholds also confirmed evidence for an association between PD and risk of schizophrenia. Furthermore, we observed no significant relationship between PD and smoking, or with other psychiatric disorders, such as attention deficit hyperactivity disorder, autism spectrum disorder, bipolar affective disorder, major depressive disorder, Alzheimer’s disease, or alcohol dependence. These results suggest that the effect of PD genetic risk on schizophrenia risk may not be via alternative risk factors, such as other psychiatric disorders or smoking [29]. Only the association between PD and cannabis use was significant at a threshold level (5 × 10^−8^), which was not replicated when the weighted median method was used (*p* > 0.05). Although a recent study suggested the potential connection between cannabis use and schizophrenia [30], our results did not support the mediating effect of other mechanisms between PD and schizophrenia.

**Figure 3:**
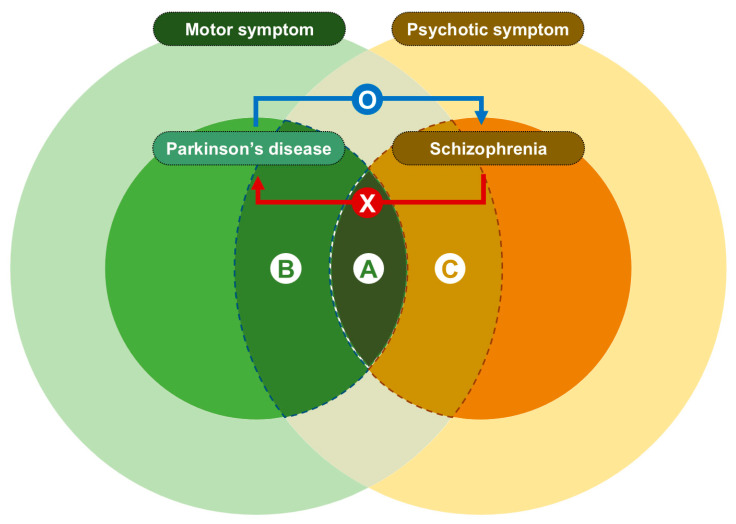
Schematic illustration of the association between Parkinson’s disease and schizophrenia from two-sample Mendelian randomization. The blue arrow indicates the direction of causal relationship between Parkinson’s disease and schizophrenia. The red arrow indicates an insignificant causal relationship between in reverse direction.

PD psychosis symptoms (A and B in Figure 3) range from mild psychotic symptoms, such as illusions or referential ideas (B in Figure 3), to prominent psychotic symptoms, including vivid hallucinations or systematized delusions that fulfilled characteristic symptoms of diagnostic criteria in schizophrenia (A in Figure 3) [31,32]. The unidirectionality of the causal effect of PD on schizophrenia implies that the psychotic symptoms in PD patients, which are similar to those in schizophrenia patients (A in Figure 3), were more likely to be due to the pathophysiology of the PD itself than dopamine agonist or environmental factors (Figure 3). This directionality could imply the possibility of genetic architecture related to PD bringing out psychotic symptoms, which could be interpreted as diagnosis of schizophrenia in clinical practice. However, the reverse relationship was not demonstrated: genetic architecture of schizophrenia was not observed to bring out PD symptoms, suggesting independent pathology of psychotic symptoms in PD from that of psychotic symptoms in schizophrenia. Even though psychotic symptoms observed in PD and schizophrenia share common clinical features, our finding suggests that genetic backgrounds in PD psychotic symptoms are independent of those in schizophrenia (A in Figure 3). This finding can also be supported by previously observed differences in dopamine related neuroimages (between PD and schizophrenia) and by novel, successful antipsychotic effect of targeting serotonin pathways on PD psychosis [33,34,35]. Clozapine is an effective antipsychotic for PD psychosis that displays low affinity for the dopamine receptor, but it has a selective effect on serotonin 5-HT2A and histamine H1 receptors [36]. In addition, pimavanserin, a recently approved drug for PD psychosis, is a selective serotonin 2A receptor inverse agonist [37]. Since schizophrenia is a subset of a psychotic disorder (Figure 3) and is not identical with the whole disease entity with a psychotic symptom, careful interpretation is needed. Further MR analyses between PD and broad psychosis phenotype [38] will be helpful to elucidate the underlying cause of psychotic symptoms in PD.

Motor symptoms in schizophrenia include extrapyramidal signs or saccadic eye movements, which are frequently observed in PD [39] (A in Figure 3) and hypokinesia, retarded catatonia, excited catatonia, echo-phenomena, or catalepsy, which are inter-related motor domains in schizophrenia [40]. Our results suggest that the motor symptom mimicking PD in schizophrenia might be related to the genetic risk of PD. Previous studies in drug-naïve schizophrenia patients with parkinsonism (A in Figure 3) have described decreased dopaminergic function in the striatum [41,42]. Considering that the main pathology of PD is dopamine depletion in the nigrostriatal pathway, these findings can be in line with our results.

PD is a neurodegenerative disorder that is frequent in middle-aged or elderly people, and schizophrenia is frequently diagnosed in individuals in their twenties and thirties [43]. However, the onset of the neurodegenerative process associated with PD remains uncertain [44]. Previous neuroimaging studies suggested that the period between the beginning of pathological changes and the onset of motor symptoms in PD would last from 3 to 6 years, although a prolonged phase may exist as long as 20 to 50 years [44,45,46]. In addition, non-motor symptoms, such as depression, rapid eye movement (REM) sleep behavior, or constipation, may precede the motor symptoms by up to 20 years [44]. Thus, it may be difficult to conclude that the onset of pathological changes of PD was later than the onset of schizophrenia. However, in consideration of the rapid progression of early-onset or young-onset PD (symptoms <50 years old), with short pre-symptomatic period and natural degenerative changes in individuals, these hypotheses need further research.

Epidemiological evidence of comorbidities of PD and schizophrenia is very rare due to obstacles in diagnosis [13]. For the diagnosis of schizophrenia, the psychotic symptoms are not attributable to the effect of medication or another medical condition, including PD or anti-Parkinson medications [32]. Moreover, there is no established diagnostic biomarker for schizophrenia [47]. As a result, only a small portion of patients with PD might be diagnosed with schizophrenia in clinical settings [48]. In addition, it is difficult to exclude drug-induced parkinsonism in patients with schizophrenia who have received anti-psychotics [49]. A case report of comorbid PD and schizophrenia presented two patients with significantly decreased dopamine transporter density in the striatum on F-18-N-(3-fluoropropyl)-2β-carbomethoxy-3β-(4-iodophenyl) nortropane positron emission tomography, which indicated genuine PD rather than drug-induced parkinsonism [13]. Further development of biomarkers for PD and schizophrenia and clinical attention for possible comorbidity of these diseases are needed.

Our study has several strengths. First, we utilized TSMR analysis using accurate estimates for the association of genetic variants with the studied traits from a very large number of samples. The increased statistical power is the key benefit of using summary statistics from the GWAS consortium in TSMR, particularly for the test of effects on dichotomous outcomes, such as PD or schizophrenia. In particular, MR analysis could be a promising method when exploring the relationship between two phenotypes with relatively rare incidence, obstacles in epidemiological studies for comorbidities, or lack of definite biomarkers. Our analyses in both directions were based on such vast samples, which might enable the identification of small effects that may exist in the context of these complex phenotypes. Second, our study revealed a robust shared genetic background between PD and schizophrenia across multiple complementary methods of TSMR analysis and multiple GWAS *p*-value cutoffs. In the presence of pleiotropic effects of instrumental genetic variants, MR-Egger provides a robust causal estimate, while the IWV and VM have better power for causal inference under the “no-pleiotropy” assumption. By applying MR-PRESSO, our analysis was less likely to violate the MR assumptions. We also showed that the causal estimates of polygenic variants passing sub-thresholds were consistent with those of GWAS variants.

Several limitations of our study must be acknowledged. We could not elaborate the specific symptomatology related to PD increasing risk of schizophrenia, because there is no currently available GWAS data for specific types of PD that are differentiated by weight of non-motor symptoms and motor symptoms. It is difficult to conduct subgroup analyses and effect moderation with publicly available GWAS summary statistics [50]. Further research can elaborate the specific symptomatology associated with increased risk of schizophrenia in PD, if there is independent GWAS data for each subphenotype of PD.

## 5. Conclusions

Our results support the view that an increased genetic risk of PD could be associated with an increased risk of schizophrenia. This finding suggests that the intrinsic pathophysiology of PD, rather than anti-Parkinson medication or environmental effects, has a more weighted effect in PD psychosis. In addition to the recent success of pimavanserine in PD psychosis, our finding provides more support for the independent pathology of PD psychosis and suggests a novel perspective on future treatment development. In addition, our results support the view that the motor symptoms in schizophrenia are causally related to the pathophysiology of PD. Thus, elaborate scales used to measure motor symptoms in PD could be applied with thorough assessment to measure motor symptoms in schizophrenia. The biological mechanisms of PD and schizophrenia need to be clarified, given their shared symptomatology and their significant impact on patients’ quality of life [31]. Furthermore, additional clinical attention for possible comorbidity of these diseases is needed.

## Figures and Tables

**Table 1:** List of the SNPs associated with risk for Parkinson’s disease (PD) and their associations with risk for schizophrenia (SCZ).

SNP	Chromosome	Position (hg19)	Gene Region	Effective Allele	PD			SCZ		
					Beta	SE of Beta	*p* Value	Beta	SE of Beta	*p* Value
rs4889730	17	21717727	None	G	−0.32	0.05	2.83 × 10^−11^	−0.02	0.01	0.04
rs2736990	4	90678541	SNCA	G	0.24	0.04	5.69 × 10^−9^	0.03	0.01	1.60 × 10^−3^
rs3784847	16	61977449	CDH8	G	0.46	0.08	1.66 × 10^−9^	0.02	0.02	0.22
rs415430	17	44859144	WNT3	C	−0.29	0.05	4.50 × 10^−8^	−0.04	0.01	1.65 × 10^−3^

SNP—single nucleotide polymorphism; Beta—ln (odds ratio); SE—standard error.

**Table 2:** Two-sample Mendelian randomization results of Parkinson’s disease and other psychiatric disorders or related traits using the inverse-variance weighted regression.

Subthreshold for PD GWAS *p*-Value	5 × 10^−^^8^		5 × 10^−^^7^		5 × 10^−^^6^	
	*p*-Value	OR (95% CI)	*p*-Value	OR (95% CI)	*p*-Value	OR (95% CI)
SCZ	3.49 × 10^−^^5^	1.10 (1.05–1.15)	7.00 × 10^−^^7^	1.10 (1.06–1.14)	6.64 × 10^−^^5^	1.06 (1.03–1.09)
ADHD	0.78	0.99 (0.91–1.07)	1.00	1.00 (0.93–1.07)	0.31	1.02 (0.98–1.05)
ASD	0.07	0.92 (0.83–1.00)	0.10	0.93 (0.85–1.01)	0.10	0.93 (0.85–1.01)
BP	0.22	1.10 (0.93–1.26)	0.11	1.10 (0.97–1.23)	0.16	1.05 (0.98–1.12)
MDD	0.92	1.00 (0.89–1.10)	0.67	0.98 (0.90–1.07)	0.22	0.97 (0.91–1.02)
AD	0.67	1.03 (0.91–1.15)	0.86	0.99 (0.86–1.11)	0.39	0.98 (0.92–1.03)
Alcohol dependence	0.91	0.98 (0.68–1.28)	0.80	0.97 (0.73–1.21)	0.58	0.96 (0.81–1.11)
Smoking	0.99	1.00 (0.64–1.36)	0.46	1.21 (0.60–1.82)	0.25	1.15 (0.87–1.43)
Cannabis use	0.005	1.02 (1.01–1.03)	0.15	1.01 (0.99–1.03)	0.19	1.01 (0.99–1.02)

PD—Parkinson’s disease; GWAS—genome-wide association study; OR—odds ratio; CI—confidence interval; SCZ—schizophrenia; ADHD— attention deficit hyperactivity disorder; ASD—autism spectrum disorders; BP—bipolar disorder; MDD—major depressive disorder; AD—Alzheimer’s disease.

## Data Availability

Data sharing not applicable. No new data were created or analyzed in this study.

## Supplementary Materials

The following are available online at https://www.mdpi.com/article/10.3390/brainsci11081042/s1, Figure S1: Forest and leave-one-out plots showing association with schizophrenia for each genetic instrument of Parkinson’s disease (PD), Table S1: Genome-wide association studies used in this study, Table S2: Two-sample Mendelian randomization results of Parkinson’s disease [2] (exposure trait) and schizophrenia [3] (outcome trait) using inverse variance weighting (IVW)and weighted median (WM), Table S3: Two-sample Mendelian randomization results of schizophrenia [3] (exposure trait) and Parkinson’s disease [2] (outcome trait) using inverse variance weighting (IVW)and weighted median (WM), Table S4: Two-sample Mendelian randomization results of schizophrenia [3] (exposure trait) and Parkinson’s disease [2] (outcome trait) using MR-Egger, Table S5: Two-sample Mendelian randomization results of Parkinson’s disease [2] (exposure trait) and schizophrenia [3] (outcome trait) using MR-Egger, Table S6: Two-sample Mendelian randomization results of Parkinson’s disease [1] (exposure trait) and schizophrenia [3] (outcome trait) using IVW and WM after removing outliers that exhibit pleiotropy detected by MR-PRESSO, Table S7: Two-sample Mendelian randomization results of Parkinson’s disease [1] (exposure trait) and schizophrenia [3] (outcome trait) using MR-Egger after removing outliers that exhibit pleiotropy detected by MR-PRESSO.
