# Supplementary material for: Shared Genetic Background between Parkinson’s Disease and Schizophrenia: A Two-Sample Mendelian Randomization Study
Source: Brain Sci. 2021 Aug 6;11(8):1042. doi: 10.3390/brainsci11081042 (PMC8393703; doi:10.3390/brainsci11081042)
Supplement: Supplementary file 1 [file brainsci-11-01042-s001.zip › brainsci-1295442-supplementary.pdf]

Supplementary Information

Supplementary Figure S1. Forest and leave-one-out plots showing association with schizophrenia for each genetic instrument of Parkinson's disease (PD)

(A) Forest plot using 32 genetic instruments (PD) [1]. (B) Leave-one-out plot using 32 genetic instruments (PD) [1].

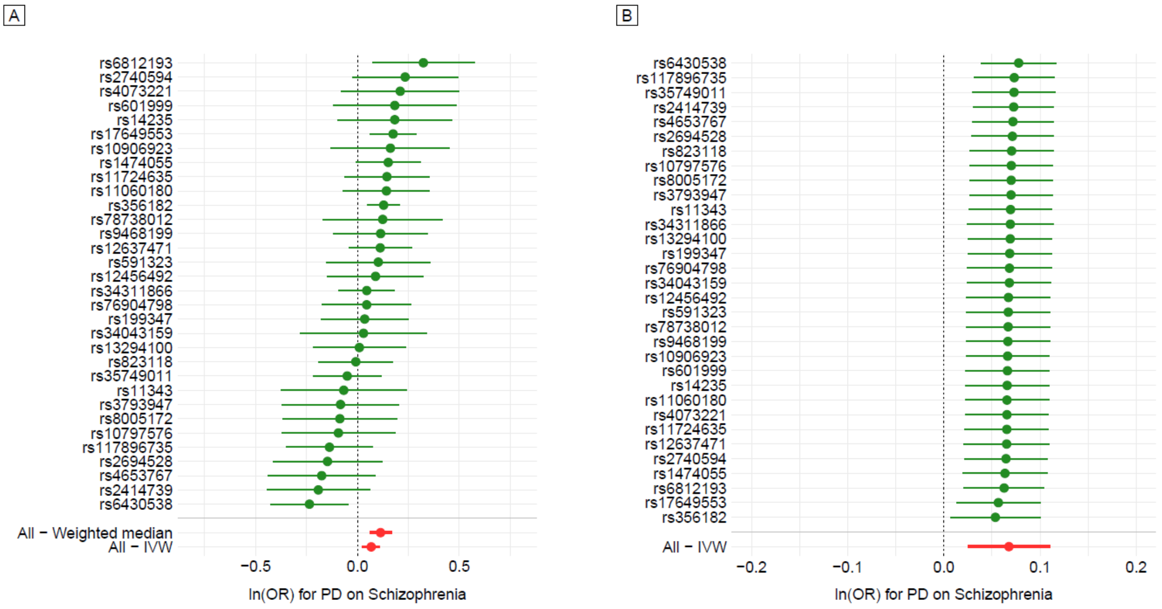

Supplementary Table S1. Genome-wide association studies used in this study.

Author	Consortium	No. of cases	No. of controls	No. of SNPs	Population	Sample size	Trait	Year
Simon-Sanchez [2]	NA	1,713	3,978	453,218	European	5,691	Parkinson's disease	2009
Chang [1]	IPDGC	6,476	302,042	-	European	308,518	Parkinson's disease	2017
Ripke [3]	PGC	35,476	46,839	9,444,231	Mixed	82,315	Schizophrenia	2014
Demontis [4]	PGC	20,183	35,191	8,047,420	European	55,374	Attention deficit hyperactivity disorder	2017
Grove [5]	PGC	18,382	27,969	6,440,259	European	46,351	Autism spectrum disorders	2017
Sklar [6]	PGC	7,481	9,250	2,427,221	European	16,731	Bipolar disorder	2011
Sullivan [7]	PGC	9,240	9,519	1,235,110	European	18,759	Major depressive disorder	2013
Lambert [8]	IGAP	17,008	37,154	7,055,882	European	54,162	Alzheimer's disease	2013
Olfson [9]	NA	1,897	1,932	917,030	Mixed	3,829	Alcohol dependence	2012
Furberg [10]	TAG	NA	NA	2,459,119	European	68,028	Cigarettes smoked per day	2010
Watanabe [11]	UK Biobank	NA	NA	9,567,039	European	126,632	Cannabis use - Ever taken cannabis	2019

SNP—single nucleotide polymorphism; IPDGC—International Parkinson's Disease Genomics Consortium; PGC—Psychiatric Genomics Consortium; IGAP—International Genomics of Alzheimer's Project; TAG—Tobacco, Alcohol and Genetics Consortium; NA—not available

Supplementary Table S2. Two-sample Mendelian randomization results of Parkinson's disease [2] (exposure trait) and schizophrenia [3] (outcome trait) using inverse variance weighting (IVW) and weighted median (WM).

		Main Mendelian randomization result (IVW)			Heterogeneity (IVW)			Sensitivity (WM)	
		**No. of SNPs**	**OR (95% CI)**	***p***	**Q (Qdf)**	**Q val**	**I^2 (%)**	**OR (95% CI)**	***p***
P threshold for PD GWAS	5×10^{-8}	4	1.10 (1.05-1.15)	3.49×10^{-5}	2.54 (3)	0.47	0	1.09 (1.02-1.15)	3.77×10^{-3}
	5×10^{-7}	5	1.10 (1.06-1.14)	7.00×10^{-7}	2.89 (4)	0.58	0	1.08 (1.02-1.14)	3.13×10^{-3}
	5×10^{-6}	14	1.06 (1.03-1.09)	6.64×10^{-5}	15.35 (13)	0.29	15	1.05 (1.01-1.10)	7.10×10^{-3}

SNPs—single nucleotide polymorphisms; PD—Parkinson's disease; GWAS—genome-wide association studies; OR—odds ratio; CI—confidence interval; Q—Cochran's heterogeneity statistic; Qdf—the degrees of freedom; I^2 –100%×(Q - Qdf)/Q [12].

Supplementary Table S3. Two-sample Mendelian randomization results of schizophrenia [3] (exposure trait) and Parkinson's disease [2] (outcome trait) using inverse variance weighting (IVW) and weighted median (WM).

		Main Mendelian randomization result (IVW)			Heterogeneity (IVW)			Sensitivity (WM)	
		**No. of SNPs**	**OR (95% CI)**	***p***	**Q (Qdf)**	**Q val**	**I^2 (%)**	**OR (95% CI)**	***p***
P threshold for SCZ GWAS	5×10^{-8}	47	1.09 (0.91-1.28)	0.31	40.66 (46)	0.70	0	1.17 (0.89-1.45)	0.19
	5×10^{-7}	66	1.07 (0.91-1.23)	0.40	62.53 (65)	0.56	0	1.15 (0.88-1.42)	0.24
	5×10^{-6}	102	1.03 (0.89-1.17)	0.72	111.30 (101)	0.23	9	1.12 (0.91-1.34)	0.24

SCZ—schizophrenia; GWAS—genome-wide association studies; SNPs—single nucleotide polymorphisms; OR—odds ratio; CI—confidence interval; Q—Cochran's heterogeneity statistic; Qdf—the degrees of freedom; I^2—100%×(Q - Qdf)/Q [12]

Supplementary Table S4. Two-sample Mendelian randomization results of schizophrenia [3] (exposure trait) and Parkinson's disease [2] (outcome trait) using MR-Egger.

		MR-Egger			Heterogeneity (Egger)			Pleiotropy (Egger)	
		**No. of SNPs**	**OR (95% CI)**	***p***	**Q (Qdf)**	**Q val**	**I^2 (%)**	**Intercept OR (95% CI)**	***p***
P threshold for SCZ GWAS	5×10^{-8}	47	0.58 (-0.01-1.18)	0.30	40.05 (45)	0.68	0	1.05 (0.97-1.13)	0.23
	5×10^{-7}	66	0.82 (0.13-1.52)	0.65	63.14 (64)	0.51	0	1.02 (0.96-1.08)	0.54
	5×10^{-6}	102	0.92 (0.24-1.60)	0.82	112.31 (100)	0.19	11	1.01 (0.96-1.06)	0.76

SCZ—schizophrenia; GWAS—genome-wide association studies; OR—odds ratio; CI, confidence interval; Q, Cochran's heterogeneity statistic; Qdf, the degrees of freedom; I^2, 100%×(Q - Qdf)/Q [12]

Supplementary Table S5. Two-sample Mendelian randomization results of Parkinson's disease [2] (exposure trait) and schizophrenia [3] (outcome trait) using MR-Egger.

		MR-Egger			Heterogeneity (Egger)			Pleiotropy (Egger)	
		**No. of SNPs**	**OR (95% CI)**	***p***	**Q (Qdf)**	**Q val**	**I^2 (%)**	**Intercept OR (95% CI)**	***p***
P threshold for PD GWAS	$5{\times}10^{-8}$	4	0.94 (0.76-1.12)	0.61	1.18 (2)	0.56	0	1.05 (0.99-1.11)	0.25
	$5{\times}10^{-7}$	5	0.98 (0.81-1.15)	0.84	2.11 (3)	0.55	0	1.03 (0.98-1.09)	0.28
	$5{\times}10^{-6}$	14	1.07 (0.94-1.21)	0.30	16.60 (12)	0.17	28	1.00 (0.97-1.03)	0.88

PD, Parkinson's disease; GWAS, genome-wide association studies; OR, odds ratio; CI, confidence interval; Q, Cochran's heterogeneity statistic; Qdf, the degrees of freedom; I^2, 100%×(Q - Qdf)/Q [12]

Supplementary Table S6. Two-sample Mendelian randomization results of Parkinson's disease[1] (exposure trait) and schizophrenia[3] (outcome trait) using IVW and WM after removing outliers that exhibit pleiotropy detected by MR-PRESSO.

		Main Mendelian randomization Result (IVW)			Heterogeneity (IVW)			Sensitivity (WM)	
		No. of SNPs	OR (95% CI)	*p*	Q (Qdf)	Q val	I^2 (%)	OR (95% CI)	*p*
P threshold for PD GWAS	5×10^{-8}	32	1.07 (1.02-1.12)	1.81×10^{-3}	45.43 (31)	0.05	32	1.12 (1.06-1.18)	2.84×10^{-5}
	5×10^{-7}	34	1.06 (1.01-1.11)	7.15×10^{-3}	54.74 (33)	0.01	40	1.12 (1.06-1.18)	3.22×10^{-5}
	5×10^{-6}	38	1.06 (1.01-1.10)	8.63×10^{-3}	58.85 (37)	0.01	37	1.12 (1.06-1.18)	3.08×10^{-5}

PD, Parkinson's disease; GWAS, genome-wide association studies; OR, odds ratio; CI, confidence interval; Q, Cochran's heterogeneity statistic; Qdf, the degrees of freedom; I^2, 100%×(Q - Qdf)/Q [12]

Supplementary Table S7. Two-sample Mendelian randomization results of Parkinson’s disease[1] (exposure trait) and schizophrenia[3] (outcome trait) using MR-Egger after removing outliers that exhibit pleiotropy detected by MR-PRESSO.

		**MR-Egger**			**Heterogeneity (Egger)**			**Pleiotropy (Egger)**	
		**No. of SNPs**	**OR (95% CI)**	***p***	**Q (Qdf)**	**Q val**	**I^2 (%)**	**Intercept OR (95% CI)**	***p***
P threshold for PD GWAS	5×10^{-8}	32	1.10 (1.00-1.21)	0.05	46.18 (30)	0.03	35	1.00 (0.98-1.01)	0.49
	5×10^{-7}	34	1.12 (1.01-1.23)	0.03	54.20 (32)	0.01	41	0.99 (0.98-1.00)	0.26
	5×10^{-6}	38	1.12 (1.02-1.23)	0.02	57.29 (36)	0.01	37	0.99 (0.98-1.00)	0.17

PD—Parkinson’s disease; GWAS—genome-wide association studies; OR—odds ratio; CI—confidence interval; Q—Cochran's heterogeneity statistic; Qdf—the degrees of freedom; I^2—100%×(Q - Qdf)/Q [12]

References

1. Chang, D.; Nalls, M.A.; Hallgrimsdottir, I.B.; Hunkapiller, J.; van der Brug, M.; Cai, F.; International Parkinson's Disease Genomics, C.; andMe Research, T.; Kerchner, G.A.; Ayalon, G.; et al. A meta-analysis of genome-wide association studies identifies 17 new Parkinson's disease risk loci. *Nat. Genet.* **2017**, *49*, 1511-1516.
2. Simon-Sanchez, J.; Schulte, C.; Bras, J.M.; Sharma, M.; Gibbs, J.R.; Berg, D.; Paisan-Ruiz, C.; Lichtner, P.; Scholz, S.W.; Hernandez, D.G.; et al. Genome-wide association study reveals genetic risk underlying Parkinson's disease. *Nat. Genet.* **2009**, *41*, 1308-1312.
3. Schizophrenia Working Group of the Psychiatric Genomics, C. Biological insights from 108 schizophrenia-associated genetic loci. *Nature* **2014**, *511*, 421-427.
4. Demontis, D.; Walters, R.K.; Martin, J.; Mattheisen, M.; Als, T.D.; Agerbo, E.; Belliveau, R.; Bybjerg-Grauholm, J.; Bækved-Hansen, M.; Cerrato, F.; et al. Discovery Of The First Genome-Wide Significant Risk Loci For ADHD. *bioRxiv* **2017**.
5. Grove, J.; Ripke, S.; Als, T.D.; Mattheisen, M.; Walters, R.; Won, H.; Pallesen, J.; Agerbo, E.; Andreassen, O.A.; Anney, R.; et al. Common risk variants identified in autism spectrum disorder. *bioRxiv* **2017**.
6. Psychiatric, G.C.B.D.W.G. Large-scale genome-wide association analysis of bipolar disorder identifies a new susceptibility locus near ODZ4. *Nat. Genet.* **2011**, *43*, 977-983.
7. Major Depressive Disorder Working Group of the Psychiatric, G.C.; Ripke, S.; Wray, N.R.; Lewis, C.M.; Hamilton, S.P.; Weissman, M.M.; Breen, G.; Byrne, E.M.; Blackwood, D.H.; Boomsma, D.I.; et al. A mega-analysis of genome-wide association studies for major depressive disorder. *Mol. Psychiatry* **2013**, *18*, 497-511.
8. Lambert, J.C.; Ibrahim-Verbaas, C.A.; Harold, D.; Naj, A.C.; Sims, R.; Bellenguez, C.; DeStafano, A.L.; Bis, J.C.; Beecham, G.W.; Grenier-Boley, B.; et al. Meta-analysis of 74,046 individuals identifies 11 new susceptibility loci for Alzheimer's disease. *Nat. Genet.* **2013**, *45*, 1452-1458.
9. Olfson, E.; Bierut, L.J. Convergence of genome-wide association and candidate gene studies for alcoholism. *Alcohol. Clin. Exp. Res.* **2012**, *36*, 2086-2094.
10. Tobacco; Genetics, C. Genome-wide meta-analyses identify multiple loci associated with smoking behavior. *Nat. Genet.* **2010**, *42*, 441-447.
11. Watanabe, K.; Stringer, S.; Frei, O.; Umićević Mirkov, M.; de Leeuw, C.; Polderman, T.J.C.; van der Sluis, S.; Andreassen, O.A.; Neale, B.M.; Posthuma, D. A global overview of pleiotropy and genetic architecture in complex traits. *Nat Genet* **2019**, *51*, 1339-1348.
12. Higgins, J.P.; Thompson, S.G.; Deeks, J.J.; Altman, D.G. Measuring inconsistency in meta-analyses. *BMJ* **2003**, *327*, 557-560.
